# Surgery: Its Theory and Practice

**Published:** 1898-09

**Authors:** 


**Surgery: its Theory and Practice.** By WILLIAM JOHNSON WALSHAM. Sixth Edition. Pp. ix., 846. London: J. & A. Churchill. 1897.

We heartily welcome a new edition of this valuable text-book. It contains many additions which bring it well up to date, and numerous illustrations have been added.

We are inclined to think that a work of this kind has distinctly a place of its own in the teaching of surgery. We should never recommend students to be content with the study of this book, but there are times—as during the earlier weeks of a dressership—when it is desirable to obtain as quickly as possible such a general idea of surgery as will enable the student to understand his work and to profit by it, and for this purpose, if for no other, we consider Walsham's *Surgery* invaluable. Then again it may bring to a student's mind some of the main facts of surgery, which are too apt to slip away if his attention is for long directed to other subjects. To the busy practitioner, forgetful of his early knowledge of surgery, it ought to be a very useful book.

